# Outcomes in patients with chronic uveitis: which factors matter to patients? A qualitative study

**DOI:** 10.1186/s12886-020-01388-y

**Published:** 2020-03-30

**Authors:** Aline C. Stolk-Vos, Hamasa Kasigar, Karlijn J. Nijmeijer, Tom O. Missotten, Jan J. Busschbach, Joris J. van de Klundert, Leonieke W. Kranenburg

**Affiliations:** 1 Rotterdam Ophthalmic Institute, Schiedamse Vest 160, Rotterdam, 3011 BH The Netherlands; 2 Erasmus School of Health Policy & Management, Erasmus University Rotterdam, Rotterdam, The Netherlands; 3 Department of Psychiatry, Section Medical Psychology and Psychotherapy, Erasmus Medical Center, Rotterdam, The Netherlands; 4 The Rotterdam Eye Hospital, Rotterdam, The Netherlands; 5 Prince Mohammad Bin Salman School of Business and Entrepreneurship, King Abdullah Economic City, Saudi Arabia

**Keywords:** Patient reported outcome measures (MeSH), Surveys and questionnaires (MeSH), Ophthalmology (MeSH), Quality of health care (MeSH), Qualitative research (MeSH), Quality of life (MeSH), Uveitis (MeSH)

## Abstract

**Purpose:**

Outcome measurements currently used in chronic uveitis care fail to cover the full patient perspective. The aim of this study is to develop a conceptual model of the factors that adult patients with chronic uveitis consider to be important when evaluating the impact of their disease and treatment.

**Methods:**

A qualitative study design was used. Twenty chronic uveitis patients were recruited to participate in two focus groups. Data were transcribed verbatim and analysed using thematic analysis in ATLAS.ti.

**Results:**

Coding of the transcripts resulted in a total of 19 codes divided over five themes: 1) disease symptoms and treatment; 2) diagnosis and treatment process; 3) impact on daily functioning; 4) emotional impact; and 5) treatment success factors.

**Conclusion:**

The conceptual model resulting from this study can contribute to the development of future uveitis specific measures in adults.

## Introduction

Chronic uveitis, a disease characterized by intraocular inflammations, is a complex and variable eye condition potentially leading to blindness and affecting adults in the working age group [1]. It is often treated systemically. Patients diagnosed with chronic uveitis not only have problems with the chronicity of the disease and side effects of the medication, but also with the unpredictability of inflammations, transient visual acuity, inflammatory activity changes, and sometimes unexpected complications of the disease and the medication used [2–4].

A previous review found high heterogeneity of outcome measures that are currently used for the evaluation of uveitis treatment. Common outcome measures were classified in several domains: 1) disease activities, 2) visual function, and 3) tissue damage or other disease complications. However, those clinical outcomes are limited in the extent to which they inform us on how patients experience the impact of their disease. For example, patients’ evaluation of their ability to conduct daily activities, such as reading and driving, are not included [5, 6]. As chronic uveitis can have a huge impact on health-related quality of life [2–4], currently used primary outcome measures may therefore fall short of appropriately addressing what patients consider as most important [7].

Commonly used instruments for patients with chronic uveitis are the SF-36 Health Survey [8] to measure health-related quality of life in a generic way and the 25-item National Eye Institute Visual Function Questionnaire (NEI-VFQ-25) [6] to measure quality of life in a domain specific way, i.e. vision-related quality of life. However, as these instruments are not specifically developed for the complex and variable condition chronic uveitis [9, 10], the resulting assessment may be incomplete. There is a disease specific instrument developed for uveitis, EYE-Q [11], but this instrument is meant for a paediatric population, while chronic uveitis is most prevalent in adults.

The development of an instrument for the adult population firstly requires understanding which factors chronic uveitis patients consider relevant. So far, there has been published no substantial qualitative in-depth research effort that focused on the patient perspectives on disease and treatment [7]. The aim of the current study is to develop a conceptual model of the factors that adult patients with chronic uveitis consider to be important when evaluating the impact of their disease and treatment. This conceptual model can contribute to the development of future uveitis specific measures in adults.

## Methods

### Study design

To determine the factors that patients with chronic uveitis consider important when evaluating the impact of their disease and treatment, we used a qualitative study design based on focus group discussion [12]. Such a focus group approach is recommended in several relevant guidelines like those of ISPOR [13] and the FDA [14], in order to assure that all factors of disease and treatment that patients consider important are determined.

This study is part of TopZorg, a project subsidized by the Dutch Organisation for Health Research and Development (ZonMw). TopZorg aims to stimulate scientific research on highly specialized care in non-academic hospitals. This study has been approved by the medical ethics committee METC of Erasmus Medical Center (MEC-2017-557).

### Study sample

We invited chronic uveitis patients of The Rotterdam Eye Hospital to participate in this study. To include a representative cross section of all chronic uveitis patients, patients were selected from the registries by means of stratified random sampling. Strata used were type of chronic uveitis, time since diagnose, gender and age. The inclusion criteria were 1) diagnosed with chronic uveitis [15] for more than 3 months; 2) having anterior segment uveitis, posterior segment uveitis, or panuveitis. We used the Dutch reimbursement codes 502 and 503, respectively referring to anterior segment uveitis and to posterior segment uveitis (intermediate and posterior) and panuveitis. These codes match with ICD-10 codes H20.x, H30.x and H44.1; 3) 18 years or older. We excluded patients who did not have a good command of the Dutch language. Two focus groups, one with 9 and one with 11 participants, were conducted to draw out different perspectives and generate discussion, thereby allowing each person to talk in detail about their perspective [16]. Selected patients received a letter with study information signed by their treating ophthalmologist. They were subsequently contacted by phone and invited to participate in the focus groups. Besides the selected patients, we invited the chairman of the uveitis patient association from the Dutch Eye Patient Association. The chairman met the inclusion criteria. All participants signed informed consent.

### Data collection

Focus group data were collected between February 2018 and March 2018. The focus groups took place at The Rotterdam Eye Hospital and were chaired by a moderator (HK). This moderator facilitated open exchange among participants. The moderator made use of a predefined semi-structured topic list with open-ended questions (Additional file 1) to structure the discussion and to prevent missing relevant topics. The topic list was based on a literature review and on input from representatives of the Dutch uveitis patient association. An observer (LK) was present to observe non-verbal communication and support the moderator if necessary. At the start of discussion, participants were asked to be respectful to each other, and the moderator emphasized the importance of hearing from every participant. The focus groups had a duration of 2 h, including a 15 min break. Focus groups were audio- and video recorded and transcribed verbatim.

### Data analysis

Thematic analysis was conducted applying a deductive approach to theme generation. Themes were selected based on the questions in the topic list (Additional file 1). Two researchers (LK and AS) carefully read the transcripts. Each of the two independently developed a structured analysis framework consisting of preliminary themes and codes. They compared their frameworks to reach consensus. Thereafter, two researchers (HK and AS) independently indexed the transcripts line by line according to this framework using ATLAS.ti [17]. Coders used memos for comments during coding. When coding was finished and the code ‘other’ was used, this code was renamed into a new or existing codename best reflecting the contents of the otherwise uncategorised transcripts. Coders compared their coding and discussed until consensus was achieved [18–20]. Subsequently, the framework was refined by removing, adding or combining codes in order to maximise internal homogeneity and external heterogeneity [21]. The final framework is added in Additional file 2. After coding was finished, the cohesion and inter-relations between codes were analysed and visually depicted in a map.

### Additional external validation

After conducting two focus groups we concluded that data saturation was achieved, i.e. no new information emerged in the second group. As there was discussion within the research group whether two focus groups might look insufficient to achieve data saturation, we decided to conduct an additional external validity check by asking chronic uveitis patients to reflex on the results, and test whether they consider the results to be complete. Such a validity check is a recommended method by Green & Thorogood [22]. More specifically, we presented the findings to six members of the uveitis patient division of the Dutch Eye Patient Association, asking them whether they concurred with the topics in the structured analysis matrix (Additional file 2), which of these topics they considered to be important, and to note missing topics.

## Results

### Participants

There were two focus group sessions involving 20 participants in total. The characteristics of the participants are described in Table 1.

**Table 1:** Patients’ characteristics participants’ focus group

	Focus group 1	Focus group 2	Total
N	11	9	20
Women, n (%)	7 (64)	5 (56)	12 (60)
Age in years, mean (range)	56 (32–74)	53 (38–65)	55 (32–74)
Diagnose code, n (%)			
- ICD-10 H20.x Anterior segment	5 (45)	5 (56)	10 (50)
- ICD-10 H30.x Posterior segment	4 (36)	2 (22)	6 (30)
- ICD-10 H44.1 Panuveitis	2 (18)	2 (22)	4 (20)
Years since diagnosis, median (range)	10 (3–13)	7 (1–14)	9 (1–14)

### Structure

Thematic analysis of the focus groups yielded five central themes characterising factors that patients with chronic uveitis consider to be important when evaluating the impact of their disease: 1) disease symptoms and characteristics; 2) diagnosis and treatment process; 3) impact on daily functioning; 4) emotional impact; and 5) treatment success factors. Table 2 lists those themes and underlying codes including a summary of the content.

**Table 2:** Summary of themes and codes, including examples

**Theme 1. Disease symptoms and treatment**	
Code 1.01 Symptoms: vision	Difference between patients: range from no vision to very good vision; fluctuating vision; diminishing vision; vision in darkness; floaters; colour perception; contrast; blurred vision; field of vision; one or two eyes affected; vision influenced by medication
Code 1.02 Symptoms: pain and discomfort	Differences between patients: range from no pain at all to unbearable pain; numb / mushy feeling / burning feeling; contraction of muscles; red eyes; light sensitivity; fatigue; tearing eye; dry eyes; distinction between long or short time since diagnose; not visible for social environment
Code 1.03 Comorbidity	Differences between patients; cause of symptoms unclear due to comorbidity; influence of comorbidity on stability of uveitis; differences in diagnostic trajectory due to comorbidity
Code 1.04 Medication use and side effects	Diversity in kind of medicines, types and dosages; self-initiated start of medication; wrong medication; lifelong use of medication; medication in consultation with doctor; knowledge about side effects results in calmness; side effects; long term effects of medicines; individual differences in medication preferences; (no) medication use as treatment outcome
**Theme 2. Diagnosis and treatment process**	
Code 2.01 Recognition / diagnostic process	Diagnosis after a lot of examination; start with wrong diagnosis; diagnosis by coincidence; diagnosis by other specialist; slow referrals from general practitioner to specialist; fast referrals from general practitioner to eye hospital; general practitioner / hospital / acute care unit / doctor in training is unknown with uveitis; wrong diagnosis and wrong medication; adopt uveitis in protocols
Code 2.02 Easy access to treating specialist	Time consuming to get to see own doctor; short consultation – face-to-face or by phone – saves a daypart in the hospital; unnecessary disease burden through bad accessibility of doctor (time, examinations, daily function); knowledge about uveitis is limited at acute care unit and by doctors in training; patient records are badly read at acute care unit; lack of central point of contact or coordinator; gives peace of mind if you know you can reach someone in case of emergency; self-initiated start with medication because doctor is not available
**Theme 3: Impact on daily functioning**	
Code 3.01 Employment	Differences between patients; ranging from lost their job to being fine with fulltime job; work adjusted; no responsive work environment
Code 3.02 Sports	No influence on sport; see ball too late; sports glasses; pain during exercising
Code 3.03 Mobility	Limited mobility; complaints dependent on weather conditions; can bike / cannot bike; cannot drive a car
Code 3.04 Watching TV / reading	Difficulties with reading; difficulties’ with watching TV
Code 3.05 Dependency	Need others to help travelling, small jobs in the house.; lifelong dependency of medication; dependency of glasses
Code 3.06 Relationships	Much understanding and social support; disorder is trivialized; difficulty in explaining the disease; not visible
**Theme 4: Emotional impact**	
Code 4.01 Uncertainty: inflammation or not?	Some patients clearly recognized an inflammation, others absolutely not; getting experienced in symptom recognition through the years; barrier to contact doctor because of doubts about having an inflammation; panic
Code 4.02 Uncertainty: future	Long term effects of medication; development of uveitis in future; fear of becoming blind; inheritability; fear that both eyes get affected; not getting better, only worse; or no worries about future
Code 4.03 Uncertainty: cause complaints	Cause is unknown, treatment cannot be focused on cause; differences between patients with or without underlying cause or comorbidity; more research into the cause of uveitis; different opinions about association food and symptoms; stress increase as a cause of symptoms
Code 4.04 Stress	Not being taken seriously by healthcare providers; accessibility of own doctor; barriers in daily functioning; uncertainty
**Theme 5: Treatment success factors**	
Code 5.02 Outcome improvement	Vision; quality of life
Code 5.01 Stability	Variety in the degree of stability; gladness when uveitis is stable; stability influenced by medication; stability is cycloid
Code 5.03 Shared decision making	Type of medication and side effects are important topics; doctor takes time and has knowledge; patient prepared for consultation; not always room for discussion; not own doctor following protocol no room for initiatives of patient; an intermediary such as rheumatism practitioner would be nice

### Theme 1 disease symptoms and treatment

The symptoms experienced and various treatment options were discussed at length. Patients reported symptoms related to *vision* and symptoms related to *pain and discomfort*. The extent to which they experienced symptoms depended on their personal condition and differed strongly between patients, e.g. from no vision to very good vision and from no pain at all to unbearable pain.

Further, patients experienced difficulties attributing symptoms to chronic uveitis, since most patients suffered from comorbid conditions (*comorbidity).* As symptoms and comorbidity were different among patients, *medication use and side effects* of that medication use also differed between patients. Treatments given to patients included steroids, immunotherapy and biologicals. Medication use received much attention in the discussions. Patients were especially interested in each other’s experiences with various types of medication, ways of taking medication – infuse, tablet, injection, drops - and dosage. Besides medication use, patients also mentioned surgeries and hospitalizations, however they did so only in relation to comorbidity and not to uveitis.

### Theme 2 diagnosis and treatment process

Most patients commented that it took long until they were correctly diagnosed with uveitis. This *diagnostic process* was characterized by slow referrals from the general practitioner to specialist care, many examinations - of which many were unnecessary -, and even misdiagnosis. For instance, a patient said: *“Actually, my optician discovered it by chance. He said: there is an inflammation in your eye. Then it took me a long time to finally get my primary care doctor’s permission. And, indeed, examination has shown that it was sarcoidosis”.*

Even when patients were diagnosed with uveitis, they experienced a poor *recognition* of uveitis by the general practitioner, emergency care physicians, and ophthalmology residents in cases where their own specialist was not available. This poor recognition resulted in inadequate examinations and medication prescriptions or in long time to treatment, as is illustrated by the following quote: *“And then you get there at the emergency department. And then you get all kinds of examinations with which you are even worse off. Sometimes also with medication that are of no use. When I get to my own ophthalmologist, I have the correct diagnosis and the right medication within five minutes, and I am done within five minutes”.*

Further, patients reported that they experienced difficulties in reaching their own uveitis specialist. They experienced the limited *accessibility* as an unnecessary disease burden. *“That you are in direct contact with him [own uveitis specialist], [...] you just want to be able to act quickly and now you are actually stopped by how it is organized.“.*

### Theme 3 impact on daily functioning

Patients varied strongly in the impact chronic uveitis had on their daily function, including activities such as *employment, sports, mobility, and watching TV or reading.* For example, one patient reported to have lost her job because of chronic uveitis, by contrast, another patient reported to do fine with her fulltime job. Further, patients discussed different patterns of *dependency* including dependency on other people, lifelong dependency on medication, and dependency on devices. To illustrate, one patient said: *“Yes, even if you just arrived in southern France and you have to say [to your spouse] the next morning: [we have to] go back again, because I have to go to Rotterdam. That has happened to me often”.*

Further, the impact on daily functioning depends on support patients experience within *relationships*. Some patients experienced much understanding from their social environment, while others felt that their environment downplayed the severity of their disease which enlarged the impact of disease burden.

### Theme 4 emotional impact

Patients highlight several emotional consequences of chronic uveitis. A main topic is the uncertainty patients experienced because of the unpredictability of the disease. We distinguished three different kinds of uncertainty*.* The first is *uncertainty about the inflammation*. Some patients could clearly recognize an inflammation, while others were unable to do so. Patients who experience difficulties in recognition made remarks like: *“But in this case: do I have it or not? And then you cross that threshold to go to a doctor. That for me is the uncertainty.”* Secondly, there is *uncertainty about the future:* the long-term effects of medication, the development of chronic uveitis, the fear of becoming blind and questions regarding inheritability. For instance, a patient said: *“That is really the rottenest thing I have, I think. Most frightening [ …*] *and uh, yes, I am afraid that my other eye, my good eye, will be like that too.”* Lastly, patients perceive *uncertainty about causes of complaints.* It involves doubt about whether it is the uveitis that causes certain complaints or whether those results from a comorbid disorder.

In addition, patients often named *stress* as an important factor. The emotional stress may be caused by the feeling of not being taken seriously by health professionals, by lack of timely access to their own ophthalmologist, by experienced barriers in daily functioning, or by the dependency caused by the chronic uveitis.

### Theme 5 treatment success factors

Treatment success factors emerged as a fifth theme. Patients perceived three main treatment success factors: 1) *outcome* – in terms of improvement in vision and/or quality of life; 2) *stability* – in terms of happiness when the uveitis is under control; and 3) the degree of *shared decision making* between patient and ophthalmologist - in terms of having enough time for consultation, sharing knowledge and experiences, and being able to exert influence on decision making on medication use. To illustrate *stability*, one patient mentioned: *“eh I also see my treatment as very successful. It has taken eight nine years, continuous bleeding, flares and inflammations in my eye. Nerves and it all. That has now completely calmed down. No bleeding, no inflammation. So, I am a happy person.”* Medication use and side effects were important topics in shared decision making. Patients noticed that shared decision making was not always there, whereas they would have liked otherwise to experience their treatment as successful.

### Cohesion between themes and codes

**Fig. 1:**
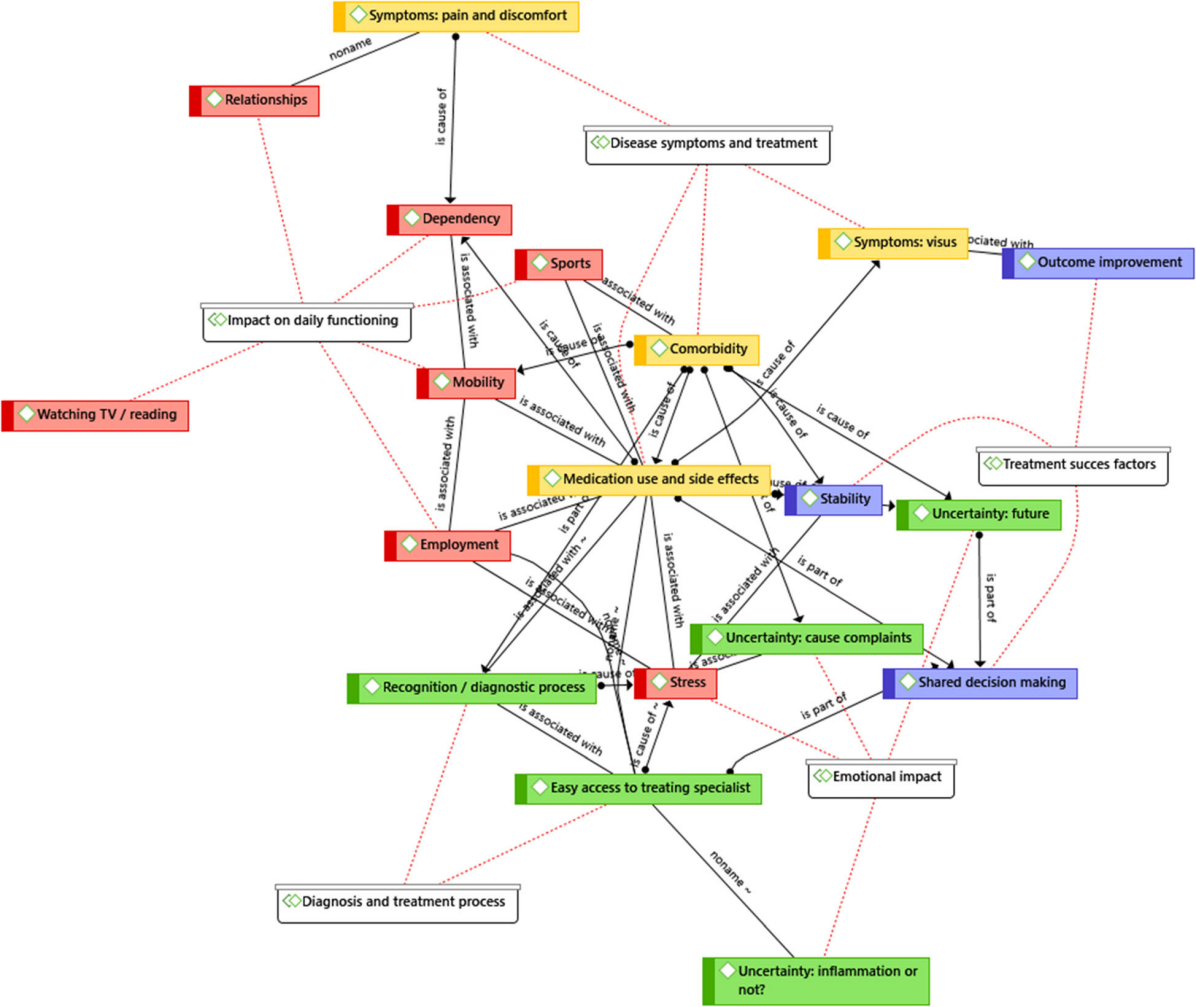
A summary model depicting the relationships between themes and codes

The cohesion and inter-relations between themes and codes is depicted in Fig. 1. *Medication and side effects* is placed in the middle indicating its central role. It is closely related to *accessibility* and *shared decision making.* This is because (questions about) medication use are an important reason for the desire for easily accessible care and an important topic during consultations according to patients. Further, it is notable that codes belonging to one and the same theme are clustered close together, which indicates the uniformity of defined themes (see Fig. 1). Lastly, we noticed that the code *stress* came up in between codes across various themes underwriting the importance of stress due to chronic uveitis in patients’ daily life.

### Additional external validation

Six members of the uveitis patient division of the Dutch Eye Patient Association took part in the additional external validity check to maximize validity (Table 3). Results were in line with our findings and no new topics came up.

**Table 3:** Patients’ characteristics of members from uveitis patient association involved in validity check

	Total
N	6
Women, n (%)	4 (67)
Age in years, mean (range)	55 (43–67)
Years since diagnosis, median (range)	12 (2–30)

## Discussion

This study shows a conceptual model with five themes that patients with chronic uveitis consider to be of importance when evaluating the impact of their disease and treatment: disease symptoms and treatment, diagnosis and treatment process, impact on daily functioning, emotional impact, and treatment success factors. Therefore, we recommend these five themes to be included in the development of future uveitis specific measures in adults.

Considering how these themes relate to the most frequently used instruments, SF-36 and VFQ-25, we notice that they only partly cover the patient perspective. The generic SF-36 may measure the theme ‘impact on daily function’ accurate yet fails to cover uveitis-specific outcomes in the themes ‘disease symptoms and treatment’, ‘diagnosis and treatment process’, specific ‘emotional impact’, and ‘treatment success factors’. Next, even though the VFQ-25 distinguishes 11 vision-related subscales, this instrument also fails to address the themes ‘diagnosis and treatment process’, ‘emotional consequences’ and some of the ‘treatment success factors’ found to be of significance for chronic uveitis by adult patients. Our findings therefore reveal that - in addition to clinical and quality of life outcomes - process factors are also relevant when measuring the impact of this complex and variable condition from a patient perspective.

Next to our main results, there are several findings worth further consideration. First, we note that access to an uveitis specialist familiar with the patient appears highly valued by patients. A trained coordinator may be beneficial to this purpose. Such a person may have added value in improving accessibility, the interdisciplinary monitoring of disease-activities, ensuring timely and accurate referral and the management of in-between-visits questions that do not require a visit to the clinic. A second finding worth highlighting is the uncertainty patients experience about short- and long-term disease outcomes. Providing information and clear communication on these matters may help patients to better prepare for the sometimes capricious disease course of chronic uveitis. A third finding for further consideration relates to the difficulties patients experience in coping with prolonged medication. Our findings suggest that better alignment with patients about risks and benefits of specific types and dosages of medication may provide patients with more control and understanding of their treatment. That may have a positive effect on how patients evaluate the outcome of their treatment, as shared decision making about medication can increase patients’ satisfaction [23]. This being said, we note that shared decision making in case of chronic uveitis can be complicated by the limited number of prospective randomized controlled trials studying the various systemic medication treatments and the complexity of the disease.

A major strength of this study was the diversity of patients who were selected by stratified sampling from patients’ records. The methods used ensured that a wide variety of chronic uveitis patients were included in the focus groups. However, we also note that by deliberately making heterogeneous groups, comparing results between subgroups becomes complex. A limitation of this study is therefore that we can only report about the heterogeneous group of chronic uveitis patients as a whole and not about subgroups e.g., patients diagnosed with ocular sarcoidosis or Birdshot retinochoroidopathy.

In conclusion, we have proposed a conceptual model containing five themes that are important when evaluating the impact of chronic uveitis in adult patients. These themes with their underlying codes can be used to develop a disease specific measurement instrument for adult chronic uveitis patients. With such an instrument patients’ disease experiences can be monitored and used to further improve the care provided and their quality of life.

## Supplementary information

**Additional file 1.** Topic list focus group.

**Additional file 2.** Final structured analysis matrix.

## Data Availability

The data that support the findings of this study are available on request from the corresponding author AS. The data are not publicly available due to containing information that could compromise research participant privacy.

## Abbreviations

NEI-VFQ-25: 25-item National Eye Institute Visual Function Questionnaire
SF-36: SF-36 Health Survey
