# Supplementary material for: Outcomes in patients with chronic uveitis: which factors matter to patients? A qualitative study
Source: BMC Ophthalmol. 2020 Mar 30;20:125. doi: 10.1186/s12886-020-01388-y (PMC7106635; doi:10.1186/s12886-020-01388-y)
Supplement: Supplementary file 1 — Additional file 1. Topic list focus group. [file 12886_2020_1388_MOESM1_ESM.docx]

# Additional file 1: Topic list focus group

1. Welcome and introduction
2. Discussion
   - Which complaints of uveitis do you experience or have you experienced? And which complaints do you experience as most stressful?
   - What impact do these complaints have on your daily life and functioning?
3. Break
4. Continue discussion
   - What do you think are success factors in the treatment?
   - What do you hope to achieve with the treatment you are undergoing or has undergone?
   - When are you satisfied with the care provided? When do you consider your treatment as successful as possible?
5. Closing
