# Supplementary material for: Outcomes in patients with chronic uveitis: which factors matter to patients? A qualitative study
Source: BMC Ophthalmol. 2020 Mar 30;20:125. doi: 10.1186/s12886-020-01388-y (PMC7106635; doi:10.1186/s12886-020-01388-y)
Supplement: Supplementary file 2 — Additional file 2. Final structured analysis matrix. [file 12886_2020_1388_MOESM2_ESM.docx]

# Additional file 2: Final structured analysis matrix

| **Themes** | **Codes** |
| --- | --- |
| Disease symptoms and treatment | Symptoms: vision |
|  | Symptoms: pain and discomfort |
|  | Comorbidity |
|  | Medication use and side effects |
| Diagnosis and treatment process | Recognition / diagnostic process |
|  | Easy access to treating specialist |
| Impact on daily functioning | Employment |
|  | Sports |
|  | Mobility |
|  | Watching TV / reading |
|  | Dependency |
|  | Relationships |
| Emotional impact | Uncertainty: inflammation or not? |
|  | Uncertainty: future |
|  | Uncertainty: cause complaints |
|  | Stress |
| Treatment success factors | Stability |
|  | Outcome improvement |
|  | Shared decision making |
